# Prevalence of Delayed Healthcare Presentation and Associated Factors in Patients with Predominantly Mild Traumatic Brain Injury: A Systematic Review and Meta-Analysis

**DOI:** 10.3390/jcm15186964

**Published:** 2026-09-08

**Authors:** Mashael Saleem Alluqmani, Talia Hussein Nooh, Saud Abdulaziz Alwayil, Shymaa Anwar Aljefri, Almuatsim Adil Al-Harrasi, Jana Omar Alghamdi, Wasan Faei Alqadi, Joud Zeid Alotaibi, Yousef Mesaed Al-Shammari, Abdallah Ali Alahmary, Bassem Yousef Sheikh

**Affiliations:** 1 College of Medicine, Taibah University, Madinah 42353, Saudi Arabia; 2 College of Medicine, King Abdulaziz University, Jeddah 21589, Saudi Arabia; talyanooh@gmail.com (T.H.N.); jaghbb881@gmail.com (J.O.A.); w_alqadi@hotmail.com (W.F.A.); jood.zeid.alotaibi@gmail.com (J.Z.A.); 3 College of Medicine, King Faisal University, Al-Hofuf 36362, Saudi Arabia; saudalwayel@gmail.com; 4 College of Medicine, King Saud bin Abdulaziz University for Health Sciences, Jeddah 22384, Saudi Arabia; shymaaljefri@gmail.com; 5 College of Medicine and Health Sciences, National University of Science and Technology, Sohar 321, Oman; almuatsimalharrasi@gmail.com; 6 Ophthalmology Department, Al-Bahar Eye Center, Ibn Sina Hospital, Ministry of Health, Kuwait City 13115, Kuwait; yousef.alnabahan@gmail.com; 7 College of Medicine, King Khalid University, Abha 61421, Saudi Arabia; dr.abdallahahalim03@gmail.com; 8 King Salman bin Abdulaziz Medical City, Madinah 42319, Saudi Arabia; consultprofsheikh@gmail.com

**Keywords:** traumatic brain injury, TBI, head trauma, brain injury, delayed presentation, late presentation, delay in care, healthcare access delay

## Abstract

**Background/Objectives:** Traumatic brain injury (TBI) is a global burden and a leading cause of death and disability worldwide. Delayed healthcare presentation is significantly associated with poor outcomes. This systematic review and meta-analysis aim to estimate the prevalence of delayed healthcare presentation in patients with traumatic brain injury, identify associated determinants, and synthesize evidence to inform strategies for early intervention and improved healthcare accessibility worldwide. **Methods:** A comprehensive electronic search was performed in PubMed and Google Scholar, including all studies published up to 1 July 2025. Eligible studies encompassed patients of any age diagnosed with mild, moderate, or severe TBI, with both the definitions of delayed presentation and TBI severity determined according to the criteria specified by each individual study. Risk of bias in the studies was assessed using the MINORS criteria for non-randomized studies and the Appraisal Tool for Cross-Sectional Studies (AXIS). All analyses were performed using OpenMeta [Analyst] software. **Results:** A total of 13 studies were included in the meta-analysis. The pooled analysis of 10 studies of participants presenting to healthcare after 24 h was 23.3% (95% CI 19.1–27.4%, I^2^ = 99.22%, *p* < 0.001). Across 8 studies that included participants aged 2 years or older, the pooled proportion presenting after 24 h was 18.7% (95% CI 13.8–23.6%, *p* < 0.001). Five studies enrolling infants younger than 2 years yielded a pooled delayed-presentation proportion of 19.8% (95% CI 14.6–25.0%, *p* < 0.001). However, the random-effects pooled proportion of participants who presented to healthcare within 24 h of head trauma was 78.0% (95% CI 73.7–82.2%, *p* < 0.001). Substantial heterogeneity was noted across all studies. **Conclusions:** This meta-analysis estimates the prevalence and evaluates the determinants of delayed healthcare presentation in patients with predominantly mild TBI, as moderate-to-severe injuries were substantially underrepresented in the included studies. Our results report several factors that contributed to the delayed presentation of TBI, including geographic, socioeconomic, and clinical factors. Additionally, it demonstrates the clinical characteristics of TBI involving timing, presenting symptoms, and injury mechanism.

## 1. Introduction

Traumatic brain injury (TBI) is a critical public health concern, ranking among the leading causes of death and disability worldwide, with an estimated global incidence of 349 per 100,000 person-years [1,2]. Its high prevalence, acute management costs, and long-term consequences, including an estimated lifetime risk affecting nearly half of the global population, place a substantial financial burden on healthcare systems in both high-income countries (HICs) and low- and middle-income countries (LMICs) [3,4]. TBI, sometimes referred to as a brain injury, head injury, or concussion, is defined as an acute disorder that occurs when external mechanical forces, such as a bump, blow, or jolt to the head, or events like falls and assaults, disrupt normal brain function [5,6].

Delayed healthcare presentation in TBI patients is a major concern, particularly in LMICs. A systematic review identified prehospital, neurosurgical, imaging, and surgical delays as critical bottlenecks [7]. Evidence from Nigeria shows that over 80% of primary care referrals were delayed due to referral pathways, distance, and financial barriers, while in Nepal, nearly half of TBI patients faced transfer delays exceeding one hour [8,9]. In Uganda, delayed initiation of emergency department interventions and limited access to timely neurosurgical care further contributed to poor prognosis [10]. These delays across multiple stages of the TBI care continuum are significantly associated with adverse outcomes, underscoring the urgent need for timely intervention and effective triage. By contrast, cohorts from high-income settings included in this review, such as the United Kingdom, the United States, and multinational PREDICT sites (Australia, New Zealand, UK, Ireland), generally reported lower proportions of delayed presentation (approximately 5–12%), suggesting that healthcare system infrastructure and accessibility may substantially influence presentation timing across settings. This review does not assert that delay in presentation is the sole or primary determinant of outcome; rather, it is examined as one modifiable factor among several, alongside prehospital stabilization, initial clinical severity, and timely surgical intervention.

However, despite the detailed insights provided by individual studies, the extent of delayed healthcare presentation and its multifactorial determinants, to the extent of our knowledge, remains incompletely synthesized in the literature. Moreover, while many studies report individual or localized findings, a comprehensive aggregation and meta-analytic assessment of prevalence rates and contributing factors have yet to be adequately established. This gap limits the formulation of targeted intervention strategies and policy development aimed at improving healthcare accessibility and timeliness for TBI patients.

Therefore, this systematic review and meta-analysis aim to address this evidence gap by estimating the prevalence of delayed healthcare presentation in patients with traumatic brain injury, identifying associated determinants, and synthesizing evidence to inform strategies for early intervention and improved healthcare accessibility worldwide.

## 2. Materials and Methods

### 2.1. Protocol and Registration

This systematic review and meta-analysis was prospectively registered in the International Prospective Register of Systematic Reviews (PROSPERO; registration number CRD420251101942); the full registry record can be accessed online (https://www.crd.york.ac.uk/PROSPERO/view/CRD420251101942) (accessed on 10 August 2026) Preferred Reporting Items for Systematic Reviews and Meta-Analyses (PRISMA) guidelines were followed accordingly, ensuring transparent and standardized reporting [11]. The PRISMA checklist is provided as Supplementary Materials (Table S1). This review utilized data extracted from previously published studies available from open access databases; therefore, ethical approval or informed consent was not required for this analysis.

### 2.2. Eligibility Criteria

Eligible studies encompassed patients of any age diagnosed with mild, moderate, or severe TBI, with both the definitions of delayed presentation and TBI severity determined according to the criteria specified by each individual study. The inclusion criteria mandated that studies report the prevalence of delayed healthcare presentation or its associated determinants, such as demographic, clinical, geographic, or health system-related variables. Both observational study designs, including cross-sectional, cohort, and case–control studies, and randomized controlled trials were considered. Studies were excluded if they failed to stratify TBI data, did not provide clear information on early versus delayed presentation timing, focused solely on therapeutic interventions without presentation data, or were non-original works such as reviews, editorials, qualitative-only studies, and conference abstracts. Additionally, non-English-language studies lacking translation were excluded.

### 2.3. Literature Search Strategy

A comprehensive electronic search was performed in PubMed and Google Scholar without date restrictions, including all studies published up to 1 July 2025. The search strategy used the following keywords: (“Traumatic brain injury” OR “TBI” OR “head trauma” OR “brain injury”) AND (“delayed presentation” OR “late presentation” OR “delay in care” OR “healthcare access delay” OR “delayed admission”). The “All Fields” option was used to maximize the search results, and only articles published in English were considered.

### 2.4. Screening and Data Extraction

All records obtained from the searches were imported into Rayyan website platform (Rayyan Systems Inc., Cambridge, MA, USA, The web version does not display a fixed version number; the platform was accessed on 20 January 2025) for deduplication and screening purposes [12]. Two authors independently screened the titles and abstracts, and studies deemed potentially eligible were subsequently evaluated in full text by another set of reviewers independently. Disagreements were resolved through discussion or adjudication by a third reviewer.

Data extraction was performed independently by one team of four authors and subsequently reviewed for accuracy by four other authors. A standardized data collection form specifically designed for this review was used. The extracted information encompassed study characteristics (such as author, year, country, design, sample size, and follow-up duration), patient characteristics (including age, sex, TBI severity, definition of delayed presentation, comorbidities, and socioeconomic or geographic factors), and outcomes (such as time to presentation, prevalence rates, determinants of delay, and associated healthcare system variables). Additionally, reported effect measures such as odds ratios, relative risks, confidence intervals, and *p*-values were documented, along with details regarding statistical methods, adjustments, and handling of missing data.

### 2.5. Risk of Bias and Quality Assessment

Risk of bias was assessed independently by two reviewers using the MINORS tool for nonrandomized observational, prospective, or retrospective studies (cohort, comparative, retrospective, prospective, and non-comparative). Disagreements were resolved by a third reviewer. MINORS consists of 12 items, with the first eight items applicable to all study designs and the remaining four items specific to comparative studies. Each item is scored on a scale of 0 to 2, with zero indicating it is not reported, one indicating it is reported but inadequate, and two indicating it is reported and adequate. The maximum total score is 16 for non-comparative studies and 24 for comparative studies [13]. Cross-sectional studies were assessed using the Appraisal Tool for Cross-Sectional Studies (AXIS). This tool consists of 20 items designed to address study design, reporting quality, and the risk of bias. The questions were answered with “yes”, “no”, or “don’t know” without a numerical scale for quality, as the researchers believed that summing scores can be misleading [14]. The quality assessment results are presented in Section 3.

### 2.6. Data Synthesis and Analysis

Data synthesis combined a structured narrative summary with quantitative pooling when studies were clinically and methodologically compatible. Using random-effects meta-analysis, proportions were pooled with corresponding 95% confidence intervals. Heterogeneity was quantified using Cochran’s Q and I^2^, and interpreted as low (<30%), moderate (30–60%), substantial (50–90%), or considerable (75–100%). All analyses were performed using OpenMeta [Analyst] software [15]. The subgroup analysis was pre-specified by age (<2 years vs. >2 years); additional subgroup analyses by TBI severity, delay-definition threshold, and injury mechanism were considered but not performed, as the number of studies within candidate subgroups was insufficient to yield reliable pooled estimates.

## 3. Results

### 3.1. Study Selection

**Figure 1:**
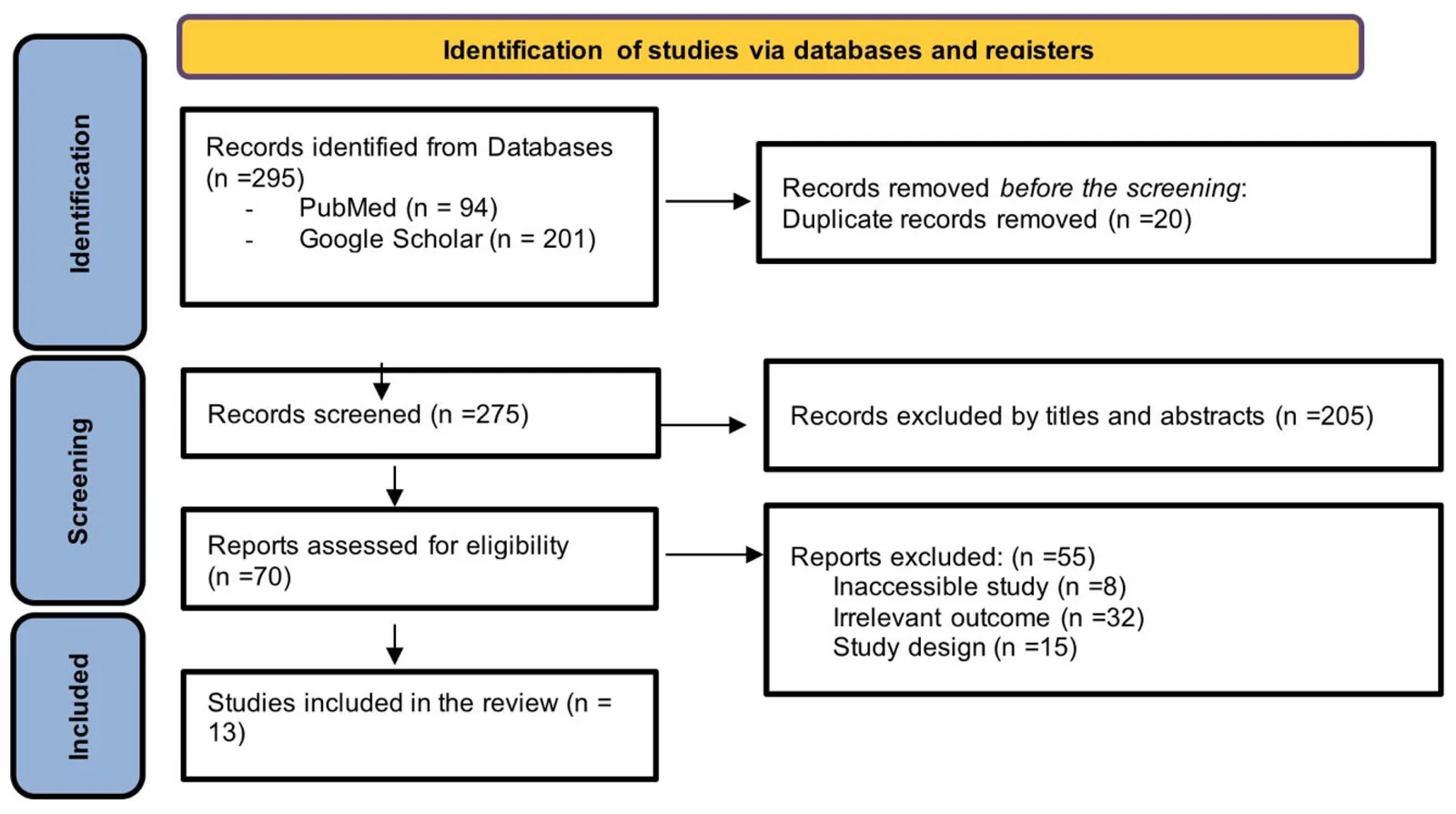
PRISMA flow diagram of study selection for the systematic review.

Studies were screened and extracted from two databases (PubMed and Google Scholar). Of the 295 studies extracted and screened, 13 that evaluated the prevalence and determinants of delayed healthcare presentation in patients with traumatic brain injury (TBI) were considered suitable for the systematic review. No randomized controlled trials (RCTs) meeting the inclusion criteria were identified; all 13 included studies were observational in design. The PRISMA flow diagram is shown in Figure 1.

### 3.2. Study Characteristics

A total of 13 included studies comprised 35,622 participants, ranging from the smallest populations of 37 and 78 to the largest population of 19,765 [16,17,18]. Age showed high heterogeneity across the study populations, from infants as young as 3 days in Sellin et al. up to older adults of 92 years in Hagos et al. [18,19]. Among the reported participants, males were the predominant gender, with approximately 65% being male and 35% being female; this aggregate figure was calculated only across the studies with reported sex distribution, excluding Khan et al. and Barrow et al., for which sex was not reported by category (Table 1).

TBI severity was assessed using the Glasgow Coma Scale, with mild head injuries (GCS 13 or 14–15) the most reported, as moderate-to-severe injuries were excluded from most studies. Only two studies included patients with moderate-to-severe injuries, identified by a GCS score of less than 14. Among them, 110 patients had moderate injuries and 57 had severe injuries [9,19]. Falls were the most prominent etiology, followed by road traffic accidents. Presenting symptoms clustered around vomiting, followed by headache and loss of consciousness, with infant cohorts additionally characterized by scalp hematoma, poor feeding, lethargy, and increasing head circumference (Table 2).

The timing of healthcare presentation among patients with TBI across the 13 studies shows significant heterogeneity but is consistently skewed toward early presentation. Five studies reported that patients presented within 24 h, with overall presentation rates of approximately 72–96% in large cohorts and 32% in a small-sample study, compared with after 24 h [16,17,20,21,25].

**Figure 2:**
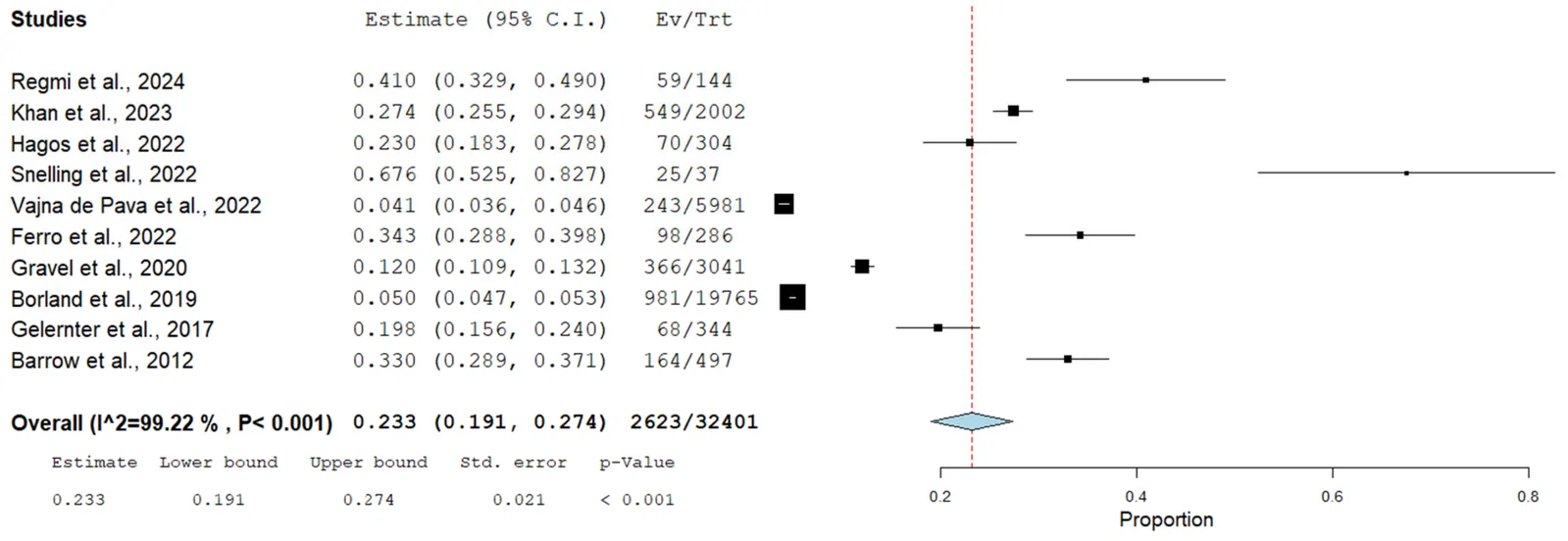
Forest plot of participants presenting with head trauma after 24 h [9,16,17,19,20,21,22,24,25,26].

**Figure 3:**
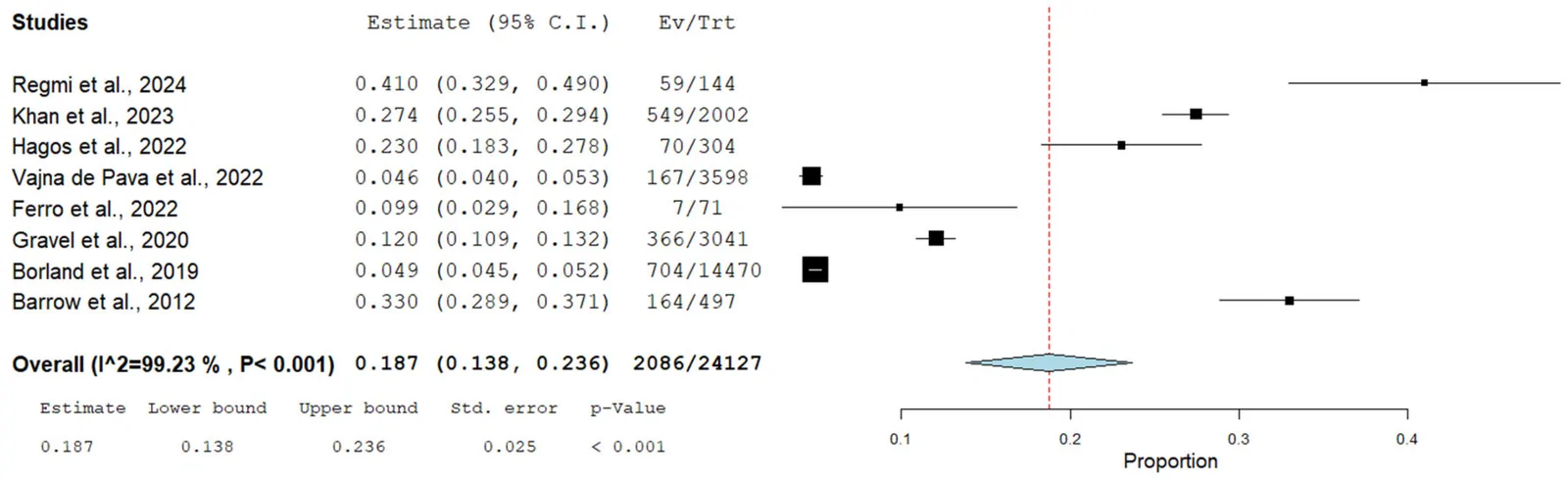
Forest plot of participants presenting with head trauma after 24 h, in those over 2 years old [9,17,19,20,21,22,24,26].

**Figure 4:**
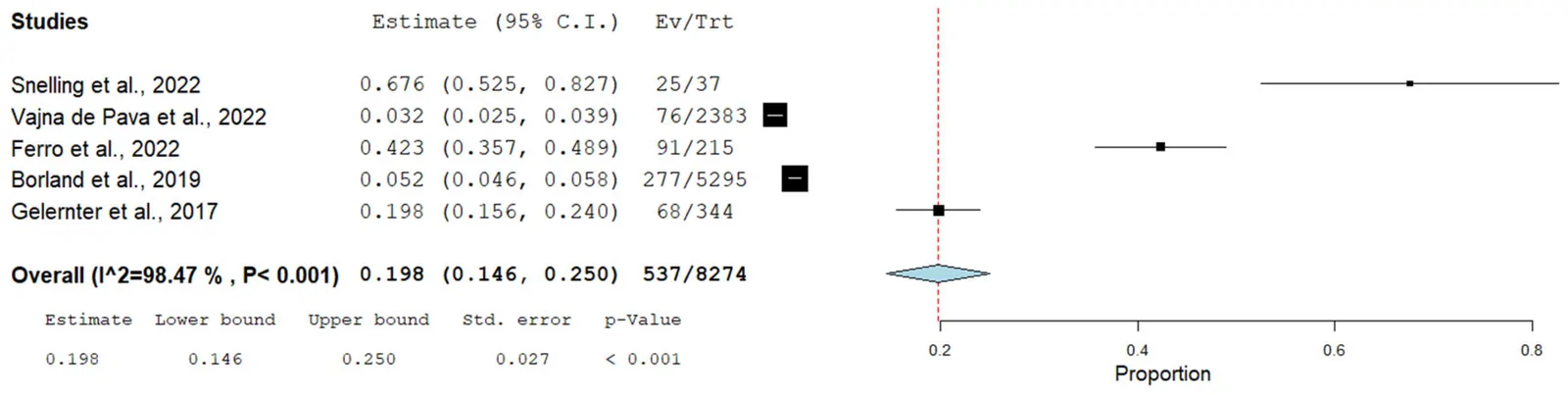
Forest plot of participants presenting with head trauma after 24 h, in those aged younger than 2 years old [16,17,21,22,25].

However, the other eight studies had diverse reporting timings. Regmi et al. reported that 59.0% (85/144) presented within 24 h, where 51.4% (74/144) of patients sought care within 1 h, while 41.0% (59/144) sought care after 24 h, of which about 14% (20/144) were over a week [9]. Hagos et al. found that 76.0% (231/304) of patients presented within 24 h, with the majority presenting within 2 to 6 h (28.9%, 88/304) [19]. Similarly, Gravel et al. showed that most were within 24 h of presentation, 88% (2675/3041), with 75.2% (2287/3041) arriving within 12 h [24]. Barrow et al. demonstrated a spread of presentation times of 4 to 12 h (38.8%), 12 to 24 h (28.2%), and after 24 h, with 12.5% within 24 to 48 h, 11.7% within 2 to 7 days, and 8.9% over a week [26]. Germann et al. used wider windows and reported 73.3% (2163/2949) presenting within a week versus 26.7% (786/2949) at 8 to 28 days [23]. Sellin’s study comprised only presentations after 24 h [18] and was therefore excluded from the pooled proportion meta-analyses (Figure 2, Figure 3 and Figure 4), as it lacked an early-presenting comparator group needed to compute a proportion of delayed versus non-delayed presentation. Ferro et al. included only children presenting more than 24 h after head injury (98/286) [22]. Hemphill et al. defined delayed presentation as ≥12 h, with all 194 patients presenting beyond this threshold [27]. All details are presented in Table 2.

The pooled analysis of ten studies included participants presenting to healthcare after 24 h, with individual study estimates (i.e., the study-level proportion of participants presenting after 24 h in each included study) ranging from 0.041 to 0.676. Germann et al. and Hemphill et al. were not included in this pooled analysis: Germann et al. used incompatible delay-definition windows (0–7 days versus 8–28 days rather than a 24-h cutoff), and Hemphill et al. defined delayed presentation as ≥12 h and enrolled only patients who had already presented beyond that threshold, precluding a comparable 24-h-threshold proportion. Large multi-centre cohorts reported low presentation proportions of 4.1% in Vajna de Pava et al., 5.0% in Borland et al., and 12.0% in Gravel et al. [17,21,24]. The overall random-effects pooled proportion was 23.3% (95% CI 19.1–27.4%), with extreme heterogeneity (I^2^ = 99.22%, *p* < 0.001) (Figure 2).

Across eight studies that included participants aged 2 years or older, the pooled proportion presenting after 24 h was 18.7% (95% CI 13.8–23.6%), with extreme heterogeneity (I^2^ = 99.23%, *p* < 0.001) (Figure 3). Five studies enrolling infants younger than 2 years yielded a pooled delayed-presentation proportion of 19.8% (95% CI 14.6–25.0%), again with extreme heterogeneity (I^2^ = 98.47%, *p* < 0.001) (Figure 4).

**Figure 5:**
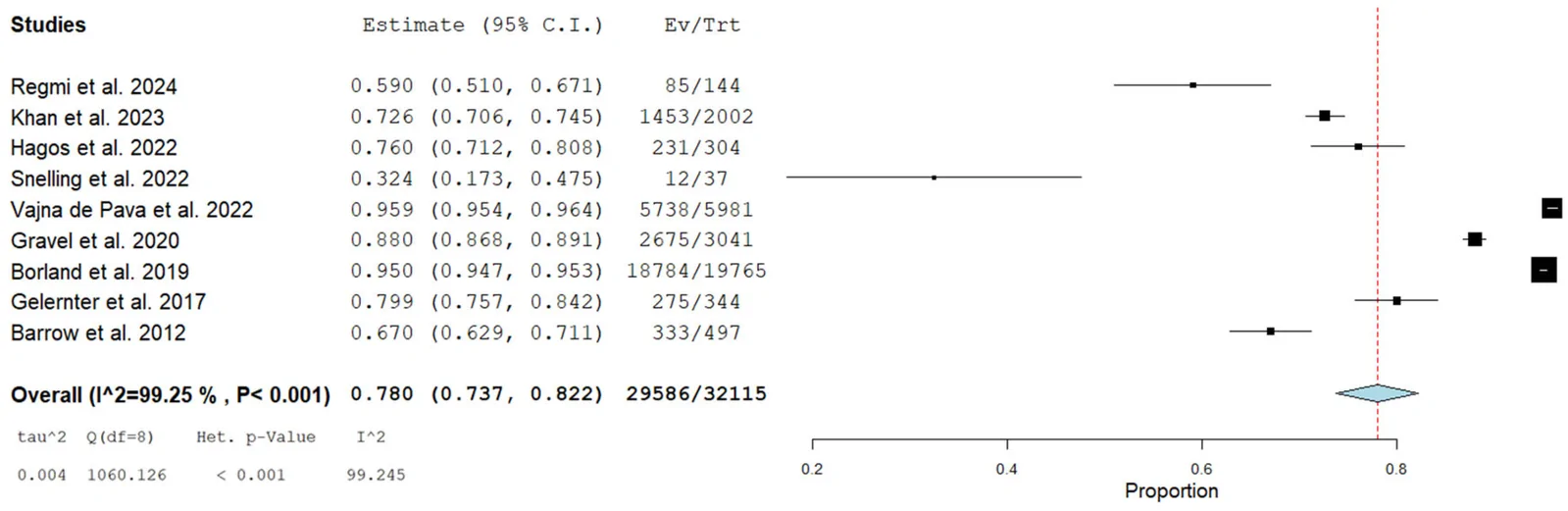
Forest plot of participants presenting with head trauma within 24 h [9,16,17,19,20,21,24,25,26].

However, the random-effects pooled proportion of participants who presented to healthcare within 24 h of head trauma was 78.0% (95% CI 73.7–82.2%). Heterogeneity was extreme (I^2^ = 99.25%, *p* < 0.001) (Figure 5). This high level of heterogeneity can be attributed to differences in age distribution, inclusion and exclusion criteria, and the various settings of the studies. Given this extreme heterogeneity, the pooled estimates presented here should be interpreted as a statistical average across clinically and demographically distinct populations (including abusive head trauma in infants, sport-related concussion in adolescents, general pediatric head injury, and adult TBI) rather than evidence of a single underlying “true” prevalence of delayed presentation.

The included studies identified several factors contributing to delayed presentation after head trauma. Regmi et al. specifically reported various geographic obstacles that contribute to delayed presentation to healthcare centers, particularly for those residing in mountainous and rural regions, along with underdeveloped infrastructure, which often leads to delays in seeking medical care [9]. Furthermore, inadequate transportation options and safety concerns make it difficult for individuals to reach hospitals. Socioeconomic risk factors were reported in Sellin et al., where delayed healthcare attendance was linked to various indicators of social vulnerability. These included factors such as being from a single-parent family, parental issues with substance abuse, recent family separations or divorces, young parenthood, and previous involvement with child protection services [18].

Clinical determinants of delayed presentation were reported in eight studies, which highlighted that this delay is often due to late-onset symptoms. Caregivers often seek medical help only after noticing visible signs like scalp swelling or hematomas, or if there are new or worsening symptoms [17,21,22,24]. In some instances, cases of abusive head trauma in children may be hidden by those responsible, leading to critical presentations when they finally do seek help [5]. Ferro et al. noted that delayed presentation was associated with older infants (aged 6 to <24 months), low-risk injury mechanisms such as falls from low heights, and a parietal hematoma location, whereas neurological symptoms and severe mechanisms were significantly less common in this group [22].

In contrast, Germann et al. found that older age and a history of sport-related concussions were associated with later presentations, while male sex, having a pre-injury baseline assessment, and a diagnosed learning disorder were linked to earlier presentations [23]. Hemphill et al. further observed that among delayed presenters, most patients with positive CT findings had concerning historical or physical features (e.g., altered mental status, focal deficits, or worsening headache), although loss of consciousness at the time of injury was not consistently reported [27].

### 3.3. Risk of Bias Assessment

The MINORS assessment of the ten non-cross-sectional (cohort or comparative) studies, and the AXIS assessment of the three cross-sectional studies (Regmi et al., Khan et al., and Hagos et al. [9,19,20]), together showed total scores that reflect a generally moderate risk of bias overall. Non-comparative cohorts had total MINORS scores ranging from 7 to 13 out of a total score of 16, with the lowest score of 7 in Snelling et al. [16], and the highest of 13 in Borland et al. [17]. The four comparative studies scored from 15 to 18 out of a total score of 24 [22,23,24,27]. Lower scores were driven by recurrent methodological limitations across studies, including absent or unclear prospective sample-size justifications, retrospective or partially retrospective data collection, incomplete follow-up or poor reporting of follow-up, and limited measures to ensure unbiased endpoint assessment. These weaknesses increase the potential for selection, information, and attrition biases. All detailed scores are presented in Table 3 and Table 4.

The details of the AXIS tool quality assessment are presented in Table 4.

## 4. Discussion

Our systematic review and meta-analysis estimated the prevalence of delayed healthcare presentation and summarized the reported determinants of delayed presentation in patients with predominantly mild TBI, since only two of the 13 included studies enrolled patients with moderate-to-severe injuries. Figure 2 demonstrates the prevalence of delayed TBI healthcare presentation, showing that the overall pooled analysis of all ages presenting late to hospital after 24 h of injury onset was 23% (*p* < 0.001). These findings suggest that approximately 1 in every 4 patients with traumatic brain injury presented late by this pooled estimate; however, this figure synthesizes studies that used differing delay-definition thresholds, ranging from 12 h to several days, and should be interpreted as a descriptive summary across heterogeneous definitions and populations rather than a single precise, generalizable rate.

To place these findings into clinical context, previous studies have shown that delayed presentation is associated with poorer functional outcomes, increased mortality, and prolonged hospitalization [28]. Quispe-Alcocer et al. reported that participants who presented late to hospital had 20% higher rates of mortality and disability compared with the other group [29]. In the study by Béavogui et al., the duration of hospital stay increased by 60% compared with early arrivals; in addition, the risk of in-hospital death was 2.6 times higher in late presenters, and this was found to be irrespective of their financial status [30]. Interestingly, the cohort study by Kwon et al. reported that clinical morbidities such as ischemic stroke, ischemic heart disease, dementia, and atrial fibrillation were 1.5–2-fold higher in patients who developed delayed intracranial hemorrhage after a negative initial CT and emergency-department discharge within 14 days [31]. In addition, TBI can result in serious long-term effects such as memory impairment, psychiatric symptoms, and increased neurodegenerative disease risk even after a mild event, which highlights the importance of early recognition and timely intervention for TBI [32].

In contrast, hospital arrival time alone does not significantly influence TBI outcomes; instead, prehospital stabilization, initial GCS, and timely surgical intervention are stronger prognostic factors [33]. Further, Quispe-Alcocer et al. stated that delayed presentation to surgery and the trauma center was associated with more negative sequelae, suggesting that time should be counted toward receiving the correct care, not solely toward hospital presentation [29]. This reflects that trauma care strategies should prioritize improving prehospital management and reducing in-hospital delays rather than strictly adhering to the ‘golden hour’ paradigm, in addition to better understanding the characteristics and factors related to presentation timing in order to improve outcomes.

As shown in Table 1, the included studies enrolled highly heterogeneous populations, ranging from infants to older adults, reflecting the broad age spectrum of patients with TBI. This heterogeneity aligns with previous epidemiological studies; for instance, Taylor et al. found that the age categories with the highest rates of TBI-related emergency department visits were ≥75 years (1701.7 per 100,000), 0–4 years (1541.1 per 100,000), and 15–24 years (1001.9 per 100,000) [34]. Furthermore, elderly age groups had the highest rates of TBI-related hospitalizations and mortality [34]. Another study by Eghzawi et al. reported that in mild and moderate TBI, age is a prognostic factor for mortality and that advanced age has a negative impact on mortality outcomes in people with moderate TBI [35].

Subgroup analyses by age (>24 h) are shown in Figure 3 and Figure 4. The pooled proportion of delayed presentation was 18.7% for participants aged 2 years or older and 19.8% for children younger than 2 years. These two pooled proportions are closely comparable, suggesting that, within our pooled data, age alone does not strongly differentiate the overall risk of delayed presentation between infants/young children and older participants; however, the clinical significance of delay likely still differs between these groups, given the more subtle and non-specific symptomatology typically seen in infants. Although the proportions were similar, infants and younger children may be more vulnerable to delayed recognition because of their limited ability to communicate symptoms and the often subtle clinical presentation of TBI. The high heterogeneity observed between studies in both age groups could be related to differences in study design, access to health care, parental awareness, socioeconomic setting, injury severity, or differences in the definition of delayed presentation. Therefore, these subgroup findings should be interpreted cautiously.

Regarding gender distribution, our systematic review demonstrates a predominance of male patients among individuals presenting with TBI. This aligns with previous epidemiological studies; for example, Eghzawi et al. reported that the majority of participants with TBI were male (approximately 61%), with 32% being female [35]. Similarly, Maleki et al. found that men were nearly four times more likely than women to be hospitalized for TBI [36]. This could be attributed to high-risk behaviors among males or occupational hazards.

Regarding TBI severity, most included studies in our review focused predominantly on mild traumatic brain injury. This is consistent with the previous literature; Cassidy et al. reported that mild TBI accounts for approximately 70–90% of all treated cases, making it the most common form of brain injury [37]. Skandsen et al. similarly demonstrated that mild TBI represents the majority of cases presenting to hospitals, emergency departments, and outpatient clinics [38]. Notably, Regmi et al. and Hagos et al. included a broader range of injury severities, encompassing moderate and severe TBI; this severity diversity reflects the wide distribution seen in clinical practice [9,19].

Regarding healthcare presentation time from injury onset, our results support the variability in the delay definition; however, across the studies, the majority of presentations were within 24 h. Importantly, despite presentation delay, significant clinical risk remains possible even after minor trauma, as demonstrated by the study of Kuppermann et al. [39]. Concerning the trauma mechanism, falls were the most commonly reported cause, and road traffic accidents (RTAs) were a major cause, especially in low- and middle-income countries, consistent with Alhabdan et al., who reported that falls and RTAs represent the leading causes of TBI in pediatric populations [40]. Notably, falls were more common in younger patients, while RTAs became proportionally more common with aging. Other reported causes included assault, sports injuries, and objects striking the head.

Different symptom presentations across age groups were also noticed. Vomiting, loss of consciousness, seizures, memory disturbance, behavioral changes, and headache were reported in adults and older children. In contrast, infants had more non-specific symptoms such as scalp hematoma, scalp swelling, increased head circumference, poor feeding, irritability, apnea, fever, and pallor, which matches the findings of Hung [41]. This difference underscores the need for greater clinical attention during assessment in this age group.

The factors that contributed to delayed hospital presentation following TBI are summarized in Table 5. They are categorized into three main domains—geographic, socioeconomic, and clinical—all of which affect care presentation in distinct ways. Our systematic review identified geographic factors as important contributors to delayed presentation, including long travel distances, mountainous terrain, limited transportation, and poorly developed infrastructure, which were consistently reported across included studies. These obstacles could prolong arrival time and reduce the likelihood of seeking early healthcare, particularly in rural areas. These findings are consistent with those reported by Shakir et al., whose systematic review highlighted a poor referral system, long travel distance, inadequacy of emergency medical services, and self-treatment practices as pre-hospital delay factors [7].

Our review also identified several socioeconomic determinants: being from a single-parent family, parental substance use, recent divorce, young parenthood, or previous involvement of child protection services due to violence were more likely to delay care-seeking. These findings emphasize that socioeconomic factors play a role in TBI presentation timing, aligning with previous studies. Kwon et al. revealed that patients insured through Medicaid were associated with delayed TBI presentation [31]. A large PREDICT cohort, for example, reported that approximately 5% of children with head injuries presented after 24 h, and delayed presenters had higher rates of clinically important traumatic brain injury on CT imaging. Although the overall proportion reported in PREDICT was lower than the proportion observed in our younger-than-2 subgroup, that study similarly emphasized that delayed presentation, while relatively uncommon, may be clinically significant and associated with more serious injuries [17]. It should be noted that the geographic determinants described above are drawn from a single study (Regmi et al. [9]), and the socioeconomic determinants are drawn from a single study (Sellin et al. [18]); these findings should therefore be regarded as illustrative rather than convergent evidence across the broader dataset, and a dedicated review targeting socioeconomic determinants of TBI presentation timing would be a valuable complement to the present analysis.

Moreover, previous studies on abusive head trauma have reported that delayed presentation may be more common in younger children because caregivers may not immediately recognize the subtle signs of head injury in this age group [42]. Overall, these findings underscore the need for targeted caregiver education on early warning signs of head trauma in infants and toddlers and the importance of timely medical assessment even when symptoms appear mild. Improving parental recognition and prompt healthcare-seeking behaviors may reduce delays and support earlier diagnosis and management of potentially serious injuries.

Our review identified several clinical factors associated with delayed presentation following TBI. These included late-onset or subtle symptoms that may be underestimated initially, leading to delayed presentation; many caregivers present only after noticing visible signs or progressive symptoms. Further, intentional delay in abusive TBI should be considered. These findings are supported by previous studies: Pak et al. reported other independent factors of delayed presentation to the emergency department, such as combined subdural and epidural hemorrhage injury type, and injury mechanisms such as unintentional falls or being struck by or against objects or indoor injury [43]. Moreover, contextual and temporal factors, including injury occurring during ordinary activities, at night, or in the winter season, were associated with delayed presentation [43]. Other studies have also noted that infants and toddlers are disproportionately represented among delayed presenters, largely due to their inability to express symptoms clearly and the tendency for early signs to be nonspecific or subtle. Overall, our findings identify high-risk populations for delayed TBI presentation, which can help guide prevention and management approaches to improve outcomes. Beyond behavioral and structural determinants, emerging fluid biomarkers of TBI, such as glial fibrillary acidic protein (GFAP), ubiquitin C-terminal hydrolase-L1 (UCH-L1), and S100 calcium-binding protein B (S100B), may offer a complementary route to earlier detection and risk stratification in patients with subtle or delayed symptom onset. Integrating such biomarkers into triage pathways could help identify clinically significant injury even when presentation is delayed, aligning delayed-presentation research with evolving precision-medicine approaches to TBI care [44].

## 5. Strengths and Limitations

This systematic review and meta-analysis addresses an important evidence gap by estimating the prevalence of delayed healthcare presentation among patients with traumatic brain injury (TBI). A large, pooled sample size across multiple countries and environments was included, increasing the generalizability of the findings. Studies were selected according to PRISMA criteria, ensuring a transparent and systematic review process. Additionally, this study analyzed not only prevalence estimates but also determinants of delayed presentation, providing a comprehensive understanding of the issue.

A key limitation of this review is that the systematic literature search was restricted to PubMed and Google Scholar, without additional databases such as Embase, Scopus, or Web of Science, and to peer-reviewed articles published in English up to the date of the search; non-English-language studies without translation were excluded. This restricted search strategy, adopted because of resource constraints, may have resulted in incomplete literature capture and introduced database and language bias, a concern that is particularly relevant given this review’s own finding that geographic and infrastructural factors in low- and middle-income settings are major determinants of delay. A formal adult (≥18 years) versus pediatric (<18 years) subgroup analysis was not feasible with the available data, as several included studies (e.g., Regmi et al., Hagos et al.) reported mixed pediatric–adult age ranges (2–84 and 2–92 years, respectively) rather than data stratified at an 18-year cutoff; this represents a direction for future primary research.

In addition, most included studies demonstrated moderate methodological quality based on MINORS. Significant heterogeneity was observed due to variations in the definitions of delayed presentation. Some studies focused on specific populations, such as sport-related concussion or abusive head trauma in children, which may affect the comparability of findings and led to clinical heterogeneity, warranting cautious interpretation of the pooled analysis. Furthermore, most studies focused on mild TBI, which limits the generalizability of the findings to severe cases. Retrospective study designs were predominant, increasing the potential risk of bias. Sex was not formally examined as a candidate moderator of heterogeneity in this analysis, as sex distribution was inconsistently reported across the included studies (e.g., not reported by category in Khan et al. and Barrow et al. [20,26]), precluding a reliable sex-stratified subgroup analysis. This is a notable gap given the approximately 65% male predominance in the pooled cohort and the finding by Germann et al. that male sex was independently associated with earlier presentation; a sex-stratified analysis of presentation timing is warranted in future primary research. Publication bias was not formally assessed using funnel plots or Egger’s regression test. With only 8–10 studies contributing to each pooled estimate, such diagnostics would have limited statistical power to reliably detect asymmetry, and this omission is acknowledged as a limitation of the current analysis.

## 6. Clinical Directions and Research Implications

This research demonstrates the need for standardization of the definition of TBI delayed presentation in future studies to reduce variability. Moreover, it calls for more research on specific subgroups and moderate-to-severe TBI grades to reflect these populations accurately. Building on our specific findings, we recommend: targeted caregiver education on early, often subtle, warning signs of head injury in infants and toddlers, given the non-specific symptomatology identified in this age group; incorporation of emerging fluid biomarkers (e.g., GFAP, UCH-L1, S100B) into triage pathways to support risk stratification irrespective of presentation timing; and dedicated future systematic reviews targeting socioeconomic determinants of TBI presentation timing and the impact of delayed surgical or operative intervention specifically (using search terms such as “delayed operation” or “delayed surgery”), which were not directly captured by the present search strategy.

## 7. Conclusions

In conclusion, we performed a systematic review of 13 studies that evaluated the prevalence and the determinants of delayed healthcare presentation in patients with traumatic brain injury (TBI). Our results report several factors that contributed to the delayed presentation of traumatic brain injury, including geographic, socioeconomic, and clinical factors. This review also demonstrates the clinical characteristics of TBI involving timing, presenting symptoms, and injury mechanism.

## Figures and Tables

**Table 1:** Demographic and participant characteristics of traumatic brain injury patients presenting to the healthcare center.

Author, Year	Country	Study Design	Sample Size (n)	Age (Years), Mean ± SD	Gender (n)
Regmi et al. 2024 [9]	Nepal	Descriptive cross-sectional	144	Range: 2–84	M: 100 F: 44
Khan et al. 2023 [20]	Pakistan	Retrospective cross-sectional	2002	39.47 >16	NS
Hagos et al. 2022 [19]	Ethiopia	Cross-sectional, hospital-based	304	30.41 ± 15.68 Range: 2–92	M: 228 F: 76
Snelling et al. 2022 [16]	Australia	Retrospective cohort study	37	<2 years Median (IQR) AHT: 4.5 (2–10) mo Non-AHT: 6 (3–11) mo	M: 21 F: 16
Vajna de Pava et al. 2022 [21]	Italy	Multicentre retrospective chart review	5981	<18 Median (IQR) <24 h: 2 (1–6) >24 h: 2 (1–8)	M: 3586 F: 2395
Ferro et al. 2022 [22]	Italy	Retrospective study	286 (98 late presentation)	0–18 years	NR
Germann et al. 2021 [23]	Canada	Historical cohort study	2949	<24 h: 14.37 ± 1.45 >24 h: 14.56 ± 1.43 Range: 12–18	M: 1659 F: 1290
Gravel et al. 2020 [24]	Canada	Secondary analysis of a prospective cohort	3041	<12 h: 11.49 12–24 h: 12.74 24–48 h: 12.71 Range: 5–17.99	M: 3040 F: 1
Borland et al. 2019 [17]	Australia and New Zealand	Prospective observational study	19,765	<18	M: 12,589 F: 7176
Sellin et al. 2017 [18]	USA	Retrospective review of a prospectively collected cohort	78	<2 years 8.8 mo Range: 3 d–24 mo	M: 48 F: 28
Gelernter et al. 2017 [25]	Israel	Retrospective chart review	344	<2 years Early: 11.4 ± 5.6 mo Late: 10.5 ± 7 mo	M: 205 F: 139
Barrow et al. 2012 [26]	UK	Observational cohort study	497	>17 Age > 65: 21	NA
Hemphill et al. 1999 [27]	USA	Retrospective chart review	194	34 ± 24	M: 82 F: 112

IQR: Interquartile Range; NA: Not Applicable; NS: Not Specified; NR: Not Reported.

**Table 2:** Clinical characteristics of traumatic brain injury patients presenting to the healthcare center.

Author, Year	TBI Severity	Time to Presentation	Etiology	Presenting Symptoms
Regmi et al. 2024 [9]	GCS Mild (13–15) = 80 Moderate (9–12) = 35 Severe (3–8) = 29	0–15 min: 45/144 16–45 min: 20/144 1 h: 9/144 >1 h: 70/144 <1 d: 85/144 1–7 d: 39/144 >7 d: 20/144	Fall: 119 Motor accident: 21 Physical assault: 4	NR
Khan et al. 2023 [20]	GCS Mild only	<24 h: 1453/2002 >24 h: 549/2002	RTA—pedestrian: 96 RTA—motorcycle: 423 RTA—car: 187 Fall: 393 Assault: 128 Other: 758	Vomiting: 215 Focal deficit: 156 Seizures: 50 Amnesia: 172 Irritability/altered behavior: 200 LOC: 435
Hagos et al. 2022 [19]	GCS Mild (14–15) = 201 Moderate (9–13) = 75 Severe (3–8) = 28	<2 h: 50/304 2–6 h: 88/304 6–12 h: 54/304 12–24 h: 39/304 >24 h: 70/304	RTA: 138 Fall: 54 Assault: 86 Other: 26 (8.6%)	NR
Snelling et al. 2022 [16]	NR	<24 h: 12/37 >24 h: 25/37	AHT: 20 Accidental trauma: 15	Floppiness: 8/37 Altered level of consciousness: 12/37 Collapse: 5/37 Vomiting: 14/37 Irritability: 5/37 Lethargy: 8/37 Seizure: 14/37 Poor feeding: 7/37 Enlarging head circumference: 3/37 Apnea: 7/37 Diarrhea: 3/37 Pallor: 4/37 Fever: 2/37 Posturing: 7/37
Vajna de Pava et al. 2022 [21]	GCS Mild (14–15) = 5981	Total <24 h: 5738/5981 >24 h: 243/5981 <2 years <24 h: 2307/2383 >24 h: 76/2383 >2 years <24 h: 3431/3598 >24 h: 167/3598	Accidental fall/hit: 5147 Other: 834	Altered mental status: 112 Signs of basal fracture: 25 Signs of skull fracture: 77 Non-frontal hematoma: 505 LOC > 5 min: 126 Severe mechanism: 479 Not acting normal: 74 Vomiting: 282 Severe headache: 161
Ferro et al. 2022 [22]	GCS Mild (≥13)	>24 h: 98/286	NR	Soft scalp hematoma, mostly parietal/occipital
Germann et al. 2021 [23]	NR	0–7 d: 2163/2949 8–28 d: 786/2949	WAD: 1045 Chronic WAD: 34 PCS: 76 SRC with LOC: 193 SRC without LOC: 2166 Other: 78 None: 349 Not provided: 26	LOC: 349 Seizure: 78 Amnesia: 448
Gravel et al. 2020 [24]	Mild only	<12 h: 2287/3041 12–24 h: 388/3041 24–48 h: 366/3041	Sports/recreational: 2059 Fall: 866 Accident: 57 Assault: 43	Seizure: 3020
Borland et al. 2019 [17]	GCS Mild (14–15) only	Total <24 h: 18,784/19,765 >24 h: 981/19,765 <2 years <24 h: 5018/5295 >24 h: 277/5295 >2 years <24 h: 13,766/14,470 >24 h: 704/14,470	Fall: 13,914 Accident: 1075 Object struck head: 1286 Assault: 95	Vomiting: 3324 Headache: 4045 LOC: 2526 Amnesia: 1606 Altered consciousness: 535 Seizures: 291
Sellin et al. 2017 [18]	Mild only	All >24 h	NR	Scalp swelling: 78 Reduced oral intake; headache/irritability; lethargy: 5
Gelernter et al. 2017 [25]	GCS Mild (14–15) only 15: 285 14: 59	<24 h: 275/344 >24 h: 68/344	Fall: 301 Accident: 21	Scalp hematoma: 223
Barrow et al. 2012 [26]	GCS Mild (14–15) only	4–12 h: 193 12–24 h: 140 24–48 h: 62 48 h–7 d: 58 >7 d: 44	NR	LOC: 113 Vomiting: 64 Headache: 199 Altered level of consciousness: 93 Amnesia: 21 Seizure: 3 Signs of base-of-skull fracture: 1
Hemphill et al. 1999 [27]	GCS Mild (15)	≥12 h after injury	Falls MVC Struck by an object	Headache, nausea, dizziness, sleepiness, confusion, coma

GCS: Glasgow Coma Scale; NR: not reported; RTA: road traffic accident; AHT: abusive head trauma; LOC: loss of consciousness; PCS: post-concussion syndrome; SRC: sport-related concussion; WAD: whiplash-associated disorder; h: hours; min: minutes; d: day; MVC: motor vehicle collision.

**Table 3:** Risk of bias assessment using the MINORS tool.

Author, Year	Q1	Q2	Q3	Q4	Q5	Q6	Q7	Q8	Q9	Q10	Q11	Q12	Total Score
Snelling et al. 2022 [16]	2	0	0	2	0	1	0	2	NA	NA	NA	NA	7/16
Vajna de Pava et al. 2022 [21]	2	2	0	2	1	2	1	0	NA	NA	NA	NA	10/16
Ferro et al. 2022 [22]	2	2	0	2	1	2	1	0	2	2	2	2	18/24
Germann et al. 2021 [23]	2	2	1	2	1	2	1	0	2	2	1	1	15/24
Gravel et al. 2020 [24]	2	2	2	2	1	2	0	0	2	2	1	1	17/24
Borland et al. 2019 [17]	2	2	2	2	1	2	2	0	NA	NA	NA	NA	13/16
Sellin et al. 2017 [18]	2	1	2	2	1	2	1	0	NA	NA	NA	NA	11/16
Gelernter et al. 2017 [25]	2	1	0	2	1	1	1	0	NA	NA	NA	NA	8/16
Barrow et al. 2012 [26]	2	1	2	2	2	1	0	0	NA	NA	NA	NA	10/16
Hemphill et al. 1999 [27]	2	2	0	2	1	2	1	0	2	2	1	0	15/24

**Table 4:** Risk of bias assessment using the AXIS tool.

	Q1	Q2	Q3	Q4	Q5	Q6	Q7	Q8	Q9	Q10	Q11	Q12	Q13	Q14	Q15	Q16	Q17	Q18	Q19	Q20
Regmi	Yes	Yes	No	Yes	Yes	Yes	No	Yes	Unclear	No	Yes	Yes	Unclear	No	Yes	Yes	Yes	Yes	No	Yes
Khan	Yes	Yes	No	Yes	Yes	Yes	No	Yes	Yes	Yes	Yes	Yes	No	No	Yes	Yes	Yes	Yes	No	Yes
Hagos	Yes	Yes	Yes	Yes	Yes	Yes	Yes	Yes	Yes	Yes	Yes	Yes	No	No	Yes	Yes	Yes	Yes	No	Yes

**Table 5:** Determinants associated with delayed presentation after head trauma.

Author, Year	Geographic	Socioeconomic	Clinical
Regmi et al. 2024 [9]	Mountainous rural living; common agricultural activity (tree climbing); challenges of inadequate infrastructure; poor safety standards in construction and transportation; timely arrival at the hospital was challenging	NR	NR
Khan et al. 2023 [20]	NR	NR	NR
Hagos et al. 2022 [19]	NR	NR	NR
Snelling et al. 2022 [16]	NR	NR	In AHT cases, perpetrators may conceal the injury, so affected infants often arrive in critical condition, sometimes presenting with symptoms such as cardiac arrest
Vajna de Pava et al. 2022 [21]	NR	NR	Caregivers often present only after they see swelling/hematoma, or after new symptoms develop
Ferro et al. 2022 [22]	NR	NR	Caregivers often present after noticing a soft scalp hematoma that appears or becomes more prominent over time; neurological symptoms and severe injury mechanisms are significantly less common in late presenters; parietal hematoma location is significantly more common in delayed presentation
Germann et al. 2021 [23]	NR	NR	Being male, having a completed pre-injury baseline assessment, and having a diagnosed learning disorder were associated with early presentation; older age and self-reported previous SRCs were associated with late presentation
Gravel et al. 2020 [24]	NR	NR	Children seeking care within the first few hours often worry about intracranial bleeding, especially after an accident; those who wait over 24 h may do so due to delayed symptom onset
Borland et al. 2019 [17]	NR	NR	Some children present more than 24 h after injury with persistent or worsening head injury symptoms, with symptoms of other injuries, or because caregivers discover a scalp hematoma made more prominent with edema and liquefaction
Sellin et al. 2017 [18]	NR	Single-parent households, parental substance abuse, recent separation/divorce, adolescent parents, prior CPS involvement	Often, days after the head trauma, as soft tissue edema progresses and the caregiver notices scalp swelling
Gelernter et al. 2017 [25]	NR	NR	NR
Barrow et al. 2012 [26]	NR	NR	NR
Hemphill et al. 1999 [27]	NR	NR	Mean age of delayed presenters was 34 years (SD ± 24); most patients with positive CT findings had concerning historical or physical features (e.g., changes in mental status, focal deficits, worsening headache, confusion); one patient with a normal initial CT later deteriorated, suggesting that initially negative imaging does not guarantee the absence of evolving pathology; loss of consciousness at the time of injury was not consistently reported in patients with positive CT findings

## Data Availability

The data supporting the findings of this study are available from the corresponding author upon reasonable request.

## Supplementary Materials

The following supporting information can be downloaded at: https://www.mdpi.com/article/10.3390/jcm15186964/s1, Table S1: PRISMA 2020 Checklist [45].

## Abbreviations

The following abbreviations are used in this manuscript:

TBI	Traumatic brain injury
HIC	High-income country
LMIC	Low- and middle-income country
PROSPERO	International Prospective Register of Systematic Reviews
PRISMA	Preferred Reporting Items for Systematic Reviews and Meta-Analyses
MINORS	Methodological Index for Non-Randomized Studies
AXIS	Appraisal Tool for Cross-Sectional Studies
GCS	Glasgow Coma Scale
NR	Not reported
RTA	Road traffic accident
AHT	Abusive head trauma
LOC	Loss of consciousness
PCS	Post-concussion syndrome
SRC	Sport-related concussion
WAD	Whiplash-associated disorder
CI	Confidence interval
CT	Computed tomography
